# Female Narcissism: Assessment, Aetiology, and Behavioural Manifestations

**DOI:** 10.1177/00332941211027322

**Published:** 2021-06-22

**Authors:** Ava Green, Rory MacLean, Kathy Charles

**Affiliations:** City University of London, Department of Psychology, London, UK; Edinburgh Napier University, School of Applied Sciences, Edinburgh, UK; Nottingham Trent University, Centre for Academic Development and Quality, Nottingham, UK

**Keywords:** Female narcissism, narcissistic personality disorder, assessment, aetiology, aggression, partner violence perpetration

## Abstract

Despite putative gender differences in the expression of narcissism, prominent theories have virtually dismissed the role of females in the development and manifestation of narcissism. The contention that narcissism is a pathology of the self that may partly differ in males and females is further evident in the Diagnostic and Statistical Manual of Mental Disorders (DSM-5). The DSM-5 reports that up to 75% of those diagnosed with Narcissistic Personality Disorder (NPD) are men. Such figures suggest that the representation of narcissism as codified in the DSM-5 may only be marginally applicable to females, given its prominent focus and nature on capturing grandiose themes which closely resemble commonly masculine norms. The overemphasis on grandiose features extends to the empirical literature which defines narcissism as a normative personality trait and is widely assessed using the Narcissistic Personality Inventory (NPI), on which males obtain significantly higher scores than females. As this review will demonstrate, one limitation frequently occurring in the literature is the attempt to comprehend narcissistic manifestations in females through the lens of what has commonly been defined as narcissism (DSM/NPI). In this review, the literature concerning the diagnostic assessment and conceptualisation of narcissistic personality disorder, aetiological factors, aggression, and partner violence perpetration will be discussed in relation to the importance of gender. This is followed by a review of existing gaps in theory and research, and suggestions for fruitful directions that can aid a richer and more meaningful literature on narcissism inclusive of gender issues.

## Introduction

The term ‘narcissism’ originates from the ancient myth of Narcissus. In Greek mythology, Narcissus was known for his exceptional beauty and was desired by many women. One of his admirers was Echo, a cursed nymph only able to speak by repeating the words of others. When Narcissus discovered her love for him, he rejected her harshly whereupon she ran and hid in shame. When discovering his own reflection in a pond of water, Narcissus fell in love with the image of himself. Thereby enamoured with this image, he repeatedly tried to embrace his reflection, thinking it was real. Unable to leave the beauty of his reflection, Narcissus wasted away through neglecting to eat or drink (Pullen & Rhodes, 2008). Such an obsession on the part of Narcissus with his own self-image led psychologists to adopt his name to describe the condition whereby individuals develop a similar unhealthy and destructive (to self or others) obsession with their own image as Narcissistic Personality Disorder (NPD; American Psychiatric Association, 2013).

The diagnostic and statistical manual of mental disorders (DSM-5) lists nine essential features of pathological narcissism, as indicative of a grandiose sense of self-importance and entitlement: a need for adulation and expectation of special treatment without commensurate skills; an impaired ability to empathise with the needs and feelings of others; interpersonal exploitation and haughty behaviours; a preoccupation with fantasies of brilliance, success, power and dominance; and a belief that others are envious of them as they themselves are of other people (American Psychiatric Association, 2013). The inclusion of narcissism as a personality disorder in the DSM-5 has generated increased interest across clinical theory, psychiatric diagnosis and social/personality psychology (Cain et al., 2008), the latter of which conceptualises narcissism as a normative personality trait (Wright et al., 2013). While alternative models of narcissism do exist (e.g., evolutionary, psychodynamic, self-regulatory processing model; for a review, see Campbell & Miller, 2011), the bulk of research that conceptualises narcissism is based on a trait approach (Campbell & Miller, 2011). The preponderance of the empirical research in the social/personality field has relied heavily upon the Narcissistic Personality Inventory (NPI), which is based on the DSM-III criteria, as the main assessment indicator of narcissism (Cain et al., 2008).

The overreliance on grandiose features of narcissism is particularly problematic for understanding gender differences in the personality construct. A longstanding overrepresentation of males in the narcissism literature has led to the widely held belief that males are more narcissistic than females. The prevalence for NPD suggests that males are up to 75% more likely to be diagnosed with this disorder than females (American Psychiatric Association, 2013), and research demonstrate marked gender differences on trait narcissism consistently occurring more prevalently in males (Blinkhorn et al., 2019; Corry et al., 2008; Foster et al., 2003; Grijalva et al., 2015; Miller & Campbell, 2008; Perry & Perry, 2004; Zeigler-Hill et al., 2008; Zerach, 2016). These findings are not surprising as these apparent gender disparities are based on the grandiosity element of narcissism (NPI/DSM) which closely resembles stereotypically masculine features, including physical expressions of aggression, an excessive need for power and an authoritarian character (Barnett & Sharp, 2017).

However, with females being less likely to endorse overt narcissistic characteristics (NPI/DSM), gender differences may instead arise in the expression of narcissistic typologies and the endorsement of narcissistic items capturing the full scope of grandiosity and vulnerability. Research has consistently found the vulnerable component of narcissism to be either gender neutral (Besser & Priel, 2009; Grijalva et al., 2015; Miller et al., 2010), or with a higher female preponderance (Green et al., 2019, 2020a, 2020b; Onofrei, 2009; Pincus et al., 2009; Rohmann et al., 2012; Wolven, 2015; Wright et al., 2010). Vulnerable narcissism is marked by shyness, shame, hypersensitivity and low self-esteem (Cain et al., 2008).

It is noteworthy that some research demonstrates a narrowing of the gender gap in narcissism (NPI) due to generational changes. For instance, a meta-analysis by Twenge et al. (2008) reviewed data on gender differences in narcissism from 1992–2006 and found that males tend to exhibit higher scores of narcissism than females, but that the mean difference decreased over time. These findings were interpreted as indicative of generational increases in agentic and assertiveness traits for which females are more likely to endorse as they gain more status. These results are, however, inconsistent with the gender differences found in narcissism using a more inclusive and larger sample size (see Foster et al., 2003). Similarly, a later meta-analysis by Grijalva et al. (2015) supports the contention that there is little evidence for a narrowing of the gender gap. Their findings were based on an updated database with a large sample size (470,846 participants), and on a review of the data on gender differences in narcissism spanning three decades. Such findings provide weight to the existence of gender difference and give more credence to the claim that these differences are not a measurement artifact, but rather represent genuine differences in the latent trait.

Narcissism manifests itself differently in men and women, and these differences may adhere to gender-role expectations associated with femininity and masculinity (Carroll, 1989; Corry et al., 2008; Green et al., 2019, 2020a; Jonason & Davis, 2018; Lamkin et al., 2017; Sherry et al., 2014; [Bibr bibr98-00332941211027322] Watson et al., 1987, 1989; Webster et al., 2007). In traditional societies, biological sex differences are believed to create a division of labor through gender socialization practices, which in turn gives rise to ‘gender appropriate’ social roles. Accordingly, most gender stereotypes fall into two categories reflective of agentic characteristics (defined as dominance, assertiveness, competitiveness and need for achievement) and communal characteristics (defined as tenderness, selflessness and nurturance), the former of which has been closely correlated with the narcissistic personality and the masculine stereotype whereas the latter is more likely to be characteristic of women and the feminine stereotype (Grijalva et al., 2015).

In fact, grandiose narcissism has been related to the stereotypical masculine expression since the very inception of the personality concept as depicted in the ancient myth of Narcissus. As such, it has been suggested that the male character of Narcissus and the female character of Echo are imbued with distinct qualities that resemble the features of grandiose and vulnerable narcissism, respectively (Onofrei, 2009). The narcissistic personality in males appears to be more commonly associated with the traditional concepts of narcissism, expressed as grandiosity, exhibitionism, entitlement, and inflated self-esteem. Conversely, narcissism in females appears to more commonly reflect the feminine form displayed by Echo, characterized by shame, hypersensitivity and low self-esteem. Despite a gendered differentiation of masculine and feminine forms of narcissism being often implied, much of the psychoanalytic and empirical literature on narcissism, as this review will demonstrate, has derived from the well-documented myth of Narcissus whereas the role of Echo has been marginalised. This has lead some researchers to ask ‘Is Echo hiding in the woods?’ (Pullen & Rhodes, 2008, p. 12).

This paper provides a review of the literature on narcissism and gender, highlighting marked gender differences in the conceptualisation and assessment of narcissistic personality disorder, aetiological factors, aggression, and partner violence perpetration. This is followed by a discussion regarding the existing gaps in theory and research, and suggestions for embedding the study of gender differences in narcissism within a theoretical framework that integrates empirically and clinically derived concepts of the construct.

## Historical review of narcissistic personality disorder – A gendered construct

 In the late nineteenth century, Harvelock Ellis (1898) invoked the myth of Narcissus and coined the term ‘Narcissus-like’ to illustrate an autoerotic sexual condition in males, a condition where a person sees the self as a sexual object. With further development in psychoanalytic theory, Otto Rank (1911) wrote exclusively on narcissism, based on his studies of female patients. Rank (1911) construed narcissism as a self-admiration and vanity that was not exclusively sexual in nature. In contrast, Freud (1914/1957) denoted narcissism as a sexual perversion, a universal stage of psycho-sexual development and a component of self-preservation, as well as an indicator of a pathological character. Originally, Freud (1914/1957) claimed that females were more narcissistic than males, on the assumed basis that females were preoccupied with their physical appearance and tended to “make object choices in reference to qualities desired for the self” (Wink & Gough, 1990, p. 448). Freud (1914/1957) signified that these individuals were extraverted, aggressive, highly independent, and unable to love or commit in close relationships.

 The psychoanalyst Reich (1933/1949) developed Freud’s (1914/1957) writings in proposing the phallic-narcissistic character, describing these individuals as reacting with cold disdain, ill humour and overt aggression towards criticism. At a deeper level, these individuals were believed to suffer from profound self-doubt regarding their masculinity. As suggested by the term, Reich’s (1933/1949) view of narcissism was somewhat intertwined with ideas of masculinity, a character trait that he argued to be more observable in men given that the narcissistic individual was over-identified with the phallus. The association between narcissism and masculinity can be seen in Adler’s (1986) concept of ‘masculine protest’, a term that represented the desire to be powerful, strong and privileged, with the intention to enhance self-esteem.

The most prominent theoreticians in the conceptualisation of narcissism were Kernberg (1975) and Kohut (1977), whose divergent aetiological formulations and nosological accounts of narcissism painted vastly different clinical pictures. Kernberg’s (1975) theory of narcissism generally reflects themes of grandiosity and aggression, a pathology he believed to be a subtype of a borderline personality configuration. According to Kernberg’s theory, a pathological narcissistic self is developed by a combination of idealised and positive characteristics of the self and others, resulting in an unrealistic, but fragile self-image. To maintain this inflated self-esteem, the pathological narcissist will defensively and consciously avoid negative aspects of self and others, thereby presenting a grandiose self.

By comparison, Kohut’s (1977) formulation of narcissistic pathology is more focused on vulnerability, shame and depression. According to Kohut’s theory, the pathological narcissist develops narcissistic defences to repel feelings of inadequacy that occur when the grandiose self is not mirrored by others, or when the individual becomes consumed by their own grandiose self-expectations. These narcissistic defences involve two forms of splitting: the first form, horizontal splitting, repressively bars unacceptable self-object needs and concerns from an individual’s consciousness. The individual can thus sustain overt manifestations of grandiosity while simultaneously refusing to acknowledge or show any feelings of shame or low self-esteem. The second form, vertical splitting, uses disavowal of needs and denial, allowing conscious experiences of vulnerability to oscillate with feelings of omnipotence. Individuals who use vertical splitting display narcissistic vulnerability through fragile self-esteem, emptiness, and shame. Although considerable disagreement exists regarding a univocal definition of this personality construct, with theorists imposing their own definition, the comprehensive contributions in the works of Kernberg (1975) and Kohut (1977) meant narcissism officially emerged as a mental disorder in the publication of the third edition of the Diagnostic and Statistical manual (DSM-III; American Psychiatric Association, 1980).

Other early theorists have, perhaps expectedly, contested that clinical observations and preeminent theories of narcissism have emerged from patriarchal and phallocentric narratives that underemphasize feminine voices and overemphasise masculinity and the male syndrome (Akhar & Thomson, 1982; Philipson, 1985; Richman & Flaherty, 1988). Philipson (1985) noted that Kernberg’s (1975) and Kohut’s (1977) discoveries and observations were based on a total of 29 clinical case materials of patients presenting traits of NPD, but only five of these depicted women. Men’s disproportionate appearance in the case studies were in light of the fact that the psychiatric patients in the clinical population were predominantly women (Philipson, 1985), thus precluding the interpretation that the gender ratio is an artefact of sampling bias in clinical setting. Instead, what these findings arguably demonstrate is that the gender bias in the presentation of narcissistic pathology as defined by the DSM is understood primarily, if not exclusively, through the male perspective.

Despite this, Kernberg’s (1975) and Kohut’s (1977) theories have been treated as a pathological syndrome which embody and afflict men and women alike. This is particularly reflective in the DSM-5 not distinguishing or highlighting any possible gender disparities in the diagnostic criteria of NPD (American Psychiatric Association, 2013), and of the ostensible gender invariance in the initial construction of the most widely used measurement of narcissism (NPI; Raskin & Terry, 1988). The overrepresentation of males in clinical case vignettes when articulating narcissistic pathology has continued to be dominant in recent literature (Dimaggio, 2012; Filippini, 2005; Kealy & Ogrodniczuk, 2011; Kealy & Rasmussen, 2012; Pincus et al., 2014; Roberts & Huprich, 2012; Russ et al., 2008).

## Gender bias in the symptomatology and assessment of narcissistic personality disorder

The significant association between the NPD diagnosis and the male gender is well established in the clinical and empirical literature (Anderson et al., 2001; Fossati et al., 2005; Jane et al., 2007; Karterud et al., 2011; Perry & Perry, 2004; Richman & Flaherty,1988; Samuels et al., 2002; Stinson et al., 2008; Torgersen et al., 2001). Such findings commonly reflect a gender bias in the criteria of NPD, in that males and females are considered on the whole to exhibit the disorder differently due to gender-related symptomatology. For instance, Pulay et al. (2012) used a large, nationally representative epidemiologic survey in the general population and found the lifetime prevalence of narcissistic PD to be higher in males than in females, with an estimation that it affected 7.7% of males and 4.8% of females. Sex differences in the NPD criteria yielded significantly greater likelihood for males to endorse ‘interpersonal exploitativeness’ and ‘lack of empathy’ than women. The authors interpreted these findings as criteria which appear to be gender-role bound, and suggested that the relationship between NPD criteria and the male gender stereotype appear to be rooted in ‘early life’. Here, identification as either a man or a woman may provide strong schemas which influence subsequent perceptions and behaviours in a way that mirrors particular gender roles and the sociocultural expectations that are associated with them.

A study conducted by Lindsay et al. (2000) explored the potential for gender bias in self-reported personality disorder inventories in a clinical sample. Findings suggested that the majority of items evidencing gender bias on the inventories derived from narcissistic scales in the direction of masculinity and adaptive attributes such as self-efficacy, confidence and self-esteem. The authors concluded that existing inventories of NPD may be biased toward interpreting adaptive masculine behaviours as being an indication of maladaptive narcissistic disorder, particularly as they relate to the gender of the patient. These findings are particularly significant if considered in the context of the fact that the most widely used PD instruments on NPD are endorsed more easily by men than women, and that certain adaptive behavioural items are characterised as pathological. What this means is that personality disorder diagnostic criteria may not have the same meaning or implications for diagnosis across narcissistic male and female patients.

Given the significance of gender roles in the expression of personality disorders, other research has explored whether college students higher in masculinity or femininity were in fact more likely to display symptoms of NPD (Klonsky et al., 2002). Both gender roles and NPD were assessed via self- and peer reports. As expected, males who behaved consistently with their gender (i.e., masculinity) exhibited more narcissistic features. Contrary to expectations, though, females who also behaved consistently with their gender (i.e., femininity) exhibited more narcissistic traits. It should be noted, however, that these preliminary findings need to be interpreted with caution due to a number of limitations. These include using a non-clinical sample (only a minority of the participants met the criteria for PD), relatively weak correlations and biased assessment instruments (based on participants’ subjective understandings of masculinity and femininity). Nevertheless, despite these limitations, other research has found no gender difference in NPD expression as it relates to items of ‘interpersonal exploitativeness’, ‘arrogance’, ‘being special and unique’ and ‘being envious’ (Karterud et al., 2011).

Moreover, Hoertel et al. (2018) were interested in exploring whether sex differences in NPD symptom expression reflect true phenomenological differences between males and females, or are due to a greater overall symptom severity in one sex in particular. Their results indicated that, out of the nine NPD symptoms, significant associations were found for two specific symptoms: ‘being envious’ and ‘lack of empathy’. As such, at lower levels of NPD severity, males were more likely than females to report the item ‘lack of empathy’, and ‘being envious’ appeared to be a stronger indicator of NPD severity in males as compared to females. The authors interpreted these findings as substantial sex differences in NPD symptom expression, however they noted that these differences may also reflect sex-bias in diagnostic criteria rather than true group differences. In other words, differences found in symptom expression in males and females may, in actual fact, reflect bias in diagnostic criteria.

Thus far, the literature into gender bias in NPD suggests that gender differences may arise in the expression of narcissistic pathology and the endorsement of NPD items, more generally reflecting the male gender expression than that of females and feminine qualities. These differences in prevalence can be accounted for in terms of females identifying more with ‘Echo’ (overt vulnerability) than with ‘Narcissus’ (overt grandiosity). Indeed, the tendency for females to exhibit the more subtle, internally hidden and vulnerable expressions of narcissistic pathology seem more prominent and have been observed in the psychoanalytic literature (Onofrei, 2009; Robinson & Graham, 2004; Ronningstam, 2006). Historically, and somewhat expectedly, grandiose and vulnerable narcissism have often been described with heavily gendered vocabulary when articulating pathologies of males and females. In 1986, O’leary and Wright noted that “these types of narcissism resemble stereotypical characterisations of male and female qualities in Western culture. Men are expected to exude confidence, to be daring, and to display their power. Women are expected to be more emotionally vulnerable. Thus, the discussion and descriptions of narcissism and narcissistic character pathology may have been complicated by gender-related phenomena” (p. 331).

Although Narcissus and Echo are not mutually exclusive of gender, the association found between Narcissus and the male gender is explicit in the psychoanalytic literature, whereas that of Echo and the female gender is not. More importantly, the failure of DSM-5 criteria to explicitly recognise any differential presentations of narcissistic grandiosity and vulnerability as guiding the assessment of psychopathology has particular implications for clinical practice in males and females. This is particularly problematic in the case of females if it is grounded in the assumption that their expression of narcissism does not fit the current DSM-5 criteria of NPD. It is important to acknowledge here that, while gender differences do not imply that a person’s biologically determined sex will be predictive of their narcissistic orientation, and while there certainty exist women who fit the DSM criteria of NPD, it is evident from the above review that narcissism (DSM) more commonly refers to male pathology.

## Narcissistic personality disorder in females

The extent to which the construct and ensuing prevalence of this psychiatric disorder is, in fact, gender-biased has significant implications for the differential diagnosis and clinical treatment of men and women. For instance, a study investigating pathological narcissism and psychotherapy found that vulnerable characteristics of narcissistic patients were associated with increased treatment utilization as compared to narcissistic patients presenting grandiose characteristics (Pincus et al., 2009). These findings indicate that diagnosticians may be more likely to treat patients who present narcissistic vulnerability. This suggests a mismatch between the presentation of grandiose narcissism (i.e. the DSM definition which tends to diagnose men; Wright et al., 2013) and vulnerable narcissism (i.e. which is currently overlooked by the DSM and tends to be more prevalent in women; Grijalva et al., 2015). The question therefore remains: what is narcissism in females being diagnosed as?

Independent of any actual differences between males and females in classifications of PDs, misdiagnoses of PDs may partly contribute to the differential prevalence rates observed in males and females (Schulte & Habel, 2018). This has led to a specific acknowledgement in the DSM-5 manual stating that “Although these differences in prevalence probably reflect real gender differences in the presence of such patterns, clinicians must be cautious not to over diagnose or under diagnose certain personality disorders in females or in males because of social stereotypes about typical gender roles and behaviours” (American Psychiatric Association, 2013, p. 648). Euler et al. (2018) argued that males are more prone to be diagnosed with NPD as a result of their more grandiose appearance of narcissism, whereas a patients’ vulnerable narcissism may be unidentified or misdiagnosed as BPD, especially in females. This is particularly significant in light of the fact that females are more likely to seek treatment than males (Skodol & Bender, 2003), and diagnosticians are more likely to evaluate NPD patients when they are in a vulnerable state (Ellison et al., 2013). Such speculations resemble the biased higher prevalence of females with BPD in clinical settings, as the latter does not reflect the balanced gender distribution found in epidemiological cohorts (Paris et al., 2013).

Grilo et al. (1996) confirmed these patterns in their sample, where it was found that the NPD diagnosis was assigned only to men whereas the BPD diagnosis was assigned significantly more frequently to women. However, the authors did not acknowledge the error in clinical judgment, arguing instead that the presentation of NPD and BPD disorders may reflect extreme manifestations of gender-linked values for males and females, respectively. In other words, the higher proportion of males with NPD may reflect a ‘developmental push’ toward power, independence and control, whereas the higher proportion of females with BPD perhaps shows a ‘developmental bias’ toward interpersonal closeness and affiliation. These ideas resonate with those of Haaken (1983), who argued that early disturbances in empathy by the caregiver, and gender socialisation, more likely produces borderline conditions for women and narcissistic personality disorders for men, a conclusion suggesting that gender issues lead to significant differences in personality pathology in men and women.

A later research study by Anderson et al. (2001) found similar patterns in prevalence rates among males and females diagnosed with DSM PDs, providing further support for the above theorisations. In this study, clinicians applied narcissistic PD and antisocial PD more frequently in men, whereas dependent, histrionic and borderline PD were diagnosed more frequently in females. The authors did note however that clinicians did not perceive the diagnostic criteria as having different implications for pathology or maladaptivity across gender. In other words, clinicians did not consider the DSM PD criteria to be more (or less) maladaptive for a man than for a woman. Although this implies that the criteria sets may have the same implications for the presence of psychopathology in males and females, the clinicians did conclude women were less likely than men to have a grandiose sense of self-importance or to be physically aggressive. On the one hand, this could be suggestive of a potential gender stereotyping, but on the other hand, a number of different data sources support the existence of biological differences between sexes from which it is concluded that females are less physically aggressive than males (e.g., Schulte & Habel, 2018; Skodol & Bender, 2003).

Interestingly, research has shown that the extent to which sex bias in diagnosis may occur is influenced by the ambiguity of the case. In a sample of trainee clinicians, Braamhorst et al. (2015) presented participants with hypothetical case vignettes containing the following: non-ambiguous case histories with sufficient features of either BPD or NPD to meet the threshold for classification, and an ambiguous case containing subthreshold features of both NPD and BPD. Results showed that there was no effect of sex of patient when sufficient information was presented to correctly diagnose BPD and NPD. However, when the case presented contained subthreshold features of both disorders, participants diagnosed BPD more often in females than in males, and NPD more often in males than in females. It is evident from the literature that narcissism is a complex, intertwined and multi-layered construct, and assessing both narcissism dimensions without explicitly recognizing the gender manifestations of the two is likely to lead to problems in the classification and treatment of NPD.

## Gender differences in the aetiology of narcissism

The above review suggest that narcissistic pathology may be a clinical phenomenon that operates differently in men and women, inviting the argument that there may be divergent precursors to the development of narcissism in males and females. The onset of narcissistic disorder is commonly attributed to abuse, trauma and early dysfunctional interactions between the child and primary caregiver. For instance, Freud (1914/1957) posited that narcissism emerged through failure of empathic response from the parent (cold and distant), or conversely, through parents overly indulging the child. Subsequent clinical theories have agreed with the importance of a lack of empathy (Kohut, 1977), combined with parental overprotectiveness (Kernberg, 1975), and overindulgence (Millon, 1981) in the emergence of narcissism. In essence, these preeminent theorists suggested that inflated and grandiose self-views in adult narcissistic individuals may serve to mask their underlying feelings of inferiority and insecurity as a result of these early childhood experiences.

The biased gender dimension in the aetiology of narcissism has been recognised in the psychoanalytic literature, which has theorised that females and males may have different predispositions to the narcissistic personality due to the process through which they are socialised (Carroll, 1989). Philipson (1985) argued that narcissism emerges as a result of a failure in empathetic responses from the mother, consequently resulting in a deficient internalised structure of the self for both genders. However, the manner in which females and males develop strategies to compensate for this faulty empathy may take different forms. As described by Philipson (1985), mothers may respond to girls as an extension of *self*, but to boys as a significant *other* figure (e.g., husband). As a result, females and males adopt different psychological strategies to compensate with the same lack of an internalised self. Males will more likely establish their ‘otherness’ through expressions of grandiosity, excessive need for admiration and extreme self-centredness. Females, on the other hand, may overly invest in or identify with significant others in an attempt to recreate the relationship they seek with the mother (Philipson, 1985). Thus, early psychoanalytic observations have led to the conclusion that the development of narcissistic defences may primarily relate to the male syndrome, whereas narcissism manifest itself differently in females.

As previously discussed, differential gendered socialisation and gender-role differences has helped generate theorisations on the observed gender differences in narcissism (Carroll, 1989; Corry et al., 2008; Green et al., 2019, 2020a, 2020b; Grijalva et al., 2015; Jonason & Davis, 2018; Lukowitsky & Pincus, 2013; Onofrei, 2009; [Bibr bibr98-00332941211027322] Watson et al., 1987, 1989). In this context, gender socialisation processes might align with certain parental styles that contribute to some extent to observed gender differences in narcissism. There is a tendency for males to display more features of grandiose narcissism and females to present with vulnerable features. This may reflect how differences in parental approaches based on child gender follow in line with particular types of socialisation designed by parents to make boys more agentic (e.g., by withholding affection, aiming to make boys more independent), and to make girls more communal and caring. If this is the case, then existing gender differences would suggest parents are using parenting styles associated with grandiose narcissism more frequently with boys than with girls (Grijalva et al., 2015).

However, a review of the research into gender differences in trait narcissism and parental styles remain inconclusive due to the heavy reliance on grandiose features of narcissism. For instance, Horton et al. (2006) found significant gender differences regarding associations of parenting with ‘unhealthy’ grandiose (total NPI score after variance associated with self-esteem is partialled out). In contrast to males, unhealthy narcissism in females was associated with parental warmth and psychological control. The authors interpreted the presence of these gender disparities as reflective of gendered socialisation processes, where females may be socialised to interpersonal relationships and males to independence. Men’s relative independence may mitigate against the impact of emotional manipulation tactics and parental attempts at over-involvement, whereas women’s relative interpersonal sensitivity means they are possibly more susceptible to the emotional and psychological consequences of such tactics.

Similarly, Capron (2004) examined recalled pampering styles (e.g. overindulgence and overprotection), and their relationship with narcissism (NPI): results supported Millon’s (1981) proposition that individuals who pamper their children foster narcissistic tendencies within them, with the overall relationship stronger for women than men. However, closer observation reveals that, not only are correlations only weak to moderate, but that the major limitation with this study is the measure of parental pampering used only represents each parenting type with a single item. In contrast to Capron’s findings, Lyons et al. (2013) used an all-female sample and found recollections of low parental care to be associated with elevated scores on the NPI Entitlement/Explotativeness facet.

Addressing the limitations with the aforementioned studies and their assessment of an unidimensional inventory of narcissism, more recent research explore the developmental antecedents to grandiose and vulnerable narcissism, and how these converge or diverge for each gender. A study conducted by Mechanic and Barry (2015) found retrospective reports of positive reinforcement and involvement parenting behaviour to be positively associated with grandiose narcissism, and perceptions of inconsistent discipline correlated with vulnerable narcissism. Regression analysis showed that, when considering gender with all other variables, inconsistent approaches to discipline were the only parenting dimension that predicted unique variance in vulnerable narcissism, with a main effect also present for gender (i.e., females scoring higher).

Investigating perceived parenting styles by both mothers and fathers, Cramer (2015) found that a mother’s parenting style was related to vulnerable narcissism, whereas a father’s parenting style was associated with the presence of grandiose narcissism. For both mothers and fathers, parenting involving permissiveness and responsiveness was negatively associated with narcissism subtypes, while authoritarian parenting was positively related to narcissism subtypes. Similarly, Huxley and Bizumic (2017) found that recollections of maternal invalidation (coldness and rejection) positively predicted vulnerable narcissism for participants who experienced lower levels of paternal invalidation, whereas higher levels of paternal invalidation positively predicted grandiose narcissism.

In an attempt to clarify previously irreconcilable findings, a recent study by  Green et al. (2020b) explored parenting styles by mothers and fathers, specifically neglectful (Kohut, 1977), strict (Kernberg, 1975), and indulgent parenting (Millon, 1981). Results showed that recalled accounts of overprotectiveness by the father was a significant positive predictor of both grandiose and vulnerable narcissism in males, whereas retrospective reports of warmth parenting by the mother significantly negatively predicted unique variance in vulnerable narcissism in females. Although these findings provide credence to the theories proposed by Kernberg (1975) and Kohut (1977) in narcissistic males and females, respectively, they also shed light on the interplay between parent gender and parenting styles in the divergent expressions of narcissism in men and women. Overall, the literature in this area accentuate the importance for future research to employ a multidimensional assessment of narcissism and parenting practices by both parents in order to more comprehensively understand and disentangle the aetiology of narcissism across gender.

## Gender differences in narcissism and aggression

Given their inherently vulnerable state, narcissists are particularly prone to experience ‘injury’ to any real or imagined threat, which in turn evokes intolerable emotions of anger, humiliation and shame (Logan, 2009). Attempts to regulate and restore the narcissistic state are believed to manifest themselves in rage, expressed either as a state of intensified and explosive anger, or in a passive-aggressive manner (See Green & Charles, 2019, for a review). Although internal and underlying psychological phenomenology (e.g., fragmented sense of self, interpersonal impairment and self-esteem dysregulation) are most likely experienced by both males and females, it is likely outward expressions of narcissism would differ by gender.

Considerable empirical research has demonstrated gender differences in narcissism (NPI) with respect to self-esteem and aggression, whereby males consistently report higher on these respective domains than women (Girgis, 2006; Sprecher et al., 2013; Velotti et al., 2014; Wallace et al., 2012; Webster, 2005; Webster et al., 2007). For instance, a study conducted by Webster (2005) found that the effects of self-esteem, physical and verbal aggression scores were significantly stronger when controlling for gender. In all domains, the effects were significantly stronger for men than women. Webster (2005) interpreted these gender-based differences as being reflective of different types of domain-specific self-esteem, in that males may adopt a ‘competitive’ domain of self-esteem (superiority) whereas women’s self-esteem may be based on cooperation (social inclusion), in light of the respective domains being positively and negatively associated with behavioural aggression, respectively.

Consistent with these findings, other research found that narcissistic males had higher levels of both proactive and reactive aggression as compared to females (Wallace et al., 2012). These gender disparities were interpreted as being reflective of socialized differences regarding how narcissistic females and males respond to stressful situations, with males more likely to engage with aggression and females with ‘*other’* coping strategies (Wallace et al, 2012). In a similar study, Webster et al. (2007) found that high self-esteem instability and narcissism were associated with increased levels of physical and verbal aggression in men but not among women, with no significant gender difference when measuring attitudinal aggression (i.e., anger and hostility). In light of these findings, the authors argued that such gender differences may be due to developmental factors in which boys behave in more overt aggression (physically and verbally aggressive), whereas girls may adopt more relational aggression suggestive of manipulation, vicious rumors or social exclusion of peers.

Although the above research provides some insight into how aggression, self-esteem and narcissism manifest in distinctive ways among female and males, these findings are nevertheless exclusively based on grandiose aspects of narcissism which arguably does not allow for a broad and comprehensive understanding of narcissism as it relates to gender expressions. Research conducted by Barnett and Powell (2016) attempted to yield a more differentiated view of gender differences in narcissism as it relates to self-esteem and aggression. Narcissism was assessed by the Pathological Narcissism Inventory (PNI; Pincus et al., 2009) which captures both grandiose and vulnerable elements of narcissism. In short, it was found that amongst men, high levels of narcissism were not associated with low self-esteem that relates to high levels of physical and verbal aggression. Amongst women, however, high rates of narcissism were associated with low self-esteem, which was in turn related to high levels of physical and verbal aggression. Although a multidimensional assessment of narcissism was included, this study conceptualised narcissism as a single dimension in that the two narcissistic subtleties and their predictive pathways in relation to aggression and self-esteem was not explored. It may be conjectured, although not considered by Barnett and Powell (2016), that these gender-based differences indicate the nature of narcissistic grandiosity and vulnerability.

Taken together, initial observations in the aggression literature suggest that female violence has been characterized as indirect and subtle in nature, linked to a low self-esteem in response to aggressive behaviour whereas male violence has been typified as more overt and grandiose in nature, and as the result of responding to perceived threats to an inflated self-esteem.

## Gender differences in narcissism and intimate partner violence

Narcissistic individuals have a pathological way of dealing with those close to them due to their interpersonal impairment, with attributes such as entitlement, need for admiration, arrogance, lack of empathy and extreme sensitivity in response to criticisms creating discord in intimate relationships (Gunderson & Ronningstam, 2001; Miller et al., 2007). Despite the significant distress and pain narcissistic individuals cause to those close others, little is known about narcissism in female perpetrators. The overrepresentation of males as offenders is common in the Intimate Partner Violence (IPV) literature whereby narcissism has often been associated with men’s perpetration of IPV (Gormley & Lopez, 2010), despite research demonstrating that female offenders of IPV exhibit significantly higher clinically elevated narcissistic traits when compared to male offenders (Simmons et al., 2005), as well as being more likely to have committed acts of general violence, including IPV, during their lifespan than narcissistic men (Blinkhorn et al., 2019). In a sample of female prison inmates, Warren et al. (2002) also found NPD to be a predictor of current incarceration for violent crime including murder. Despite these alarming results, the majority of research in this area rely only on grandiose features of narcissism (or a sub-component of grandiose narcissism) when articulating the presentation of narcissism in females.

Gormley and Lopez (2010) examined the effects of gender, attachment styles, stressors and the entitlement element of grandiose narcissism toward students’ propensity to engage in psychological abuse of their intimate partners. Results indicated that narcissistic entitlement implied inclinations toward devaluing partners as a means to value the self, and that these inclinations explained a substantial portion of psychological abuse, particularly among men. It was found that men who avoided intimacy, who perceived themselves as having stressful problems, or who had an elevated sense of entitlement were most likely to psychologically abuse their partners. Women, however, were in contrast not found to be psychologically abusive except when all these conditions were present. Gormley and Lopez (2010) argued that females who feel entitled to exploit others to achieve own ends, distance themselves from intimacy and do not identify themselves as having stressful problems may be at high risk of psychological abuse perpetration. However, the findings of this study need to be cautiously interpreted as it can be argued that the partial assessment of an already unidimensional aspect of narcissism is measuring entitled individuals and not necessarily narcissistic individuals.

Research utilizing dyadic data analysis has provided some insight into the gendered nature of narcissism as it relates to the perpetration of verbal abuse. For instance, a recent study by Lamkin et al. (2017) explored narcissism (NPI) in relation to observed communication (e.g., anger, hostility) during a neutral discussion task. In short, results from a dyadic analysis (coded by observers) from a single lab visit revealed that women with higher levels of narcissism demonstrated significantly higher levels of hostile and angry communication patterns. These findings replicate other research indicating that women’s, but not men’s, narcissism significantly predicted marital trajectories over time (Lavner et al., 2016). In addressing the often brief interactions in lab-based studies, a short-term longitudinal research by Caiozzo et al. (2016) sought to address the complex nature of IPV by assessing grandiose narcissism, emotion regulation and attitudes towards aggression over a two month period. Results indicated that high levels of verbal aggression was perpetrated by both males and females who held beliefs that aggression was a justifiable response and who reported lower levels of emotion regulation.

Other dyadic research has stressed that gender is a key expression in narcissism as it relates to IPV (Ryan et al., 2008). In this study, the exploitative/entitlement factor of grandiose narcissism, vulnerable narcissism, and both partners’ aggression (i.e. physical and sexual aggression/coercion) was measured in 63 couples. Results indicated that, for women, only the exploitativeness/entitlement factor of narcissism significantly correlated with aggression (i.e. sexual coercion) in both partners. Ryan et al. (2008) argued that exploitative and entitled women may be hypersensitive to the perceived coercive behaviours of their partners. Alternatively, they may feel entitled to exert coercion and manipulation as a means to gain control over their partners. Results also indicated that gender is a key factor in narcissism due to the discrepancies in couples’ ratings of aggression, suggesting that perceptions of narcissism and aggression operated at an individual-level rather than couple-level.

Further research exploring the exploitativeness/entitlement sub-scale of narcissism in IPV has suggested that entitled and exploitative females and males may differ in their expression of aggression in intimate relationships (Southard, 2010). In this study, it was found that the exploitativeness/entitlement factor and vulnerable narcissism was only related to women’s use of specific influence tactics (i.e. bullying, supplication and disengagement), but not for men. Consistent with previous research (i.e., Ryan et al., 2008), these findings may indicate that exploitative and entitled women’s aggression may be expressed in more coercive forms.

A similar study conducted by Blinkhorn et al. (2015) explored grandiose narcissism in relation to sexually coercive behaviour in both men and females. As expected, it was found that males scored higher on the NPI and reported using more sexually coercive tactics than women did. Interestingly, these coercive tactics (i.e., emotional manipulation, sexual arousal and exploitation) were predicted by the adaptive facets of grandiose narcissism (i.e. leadership/authority and grandiose/exhibitionism). For women, the maladaptive facet of narcissism (i.e. exploitativeness/entitlement) was a stronger predictor of serious and aggressive sexual coercive behaviour than it was for males. In other words, the use of sexual coercion in females reflected the manipulative and sexually toxic aspect of narcissism, whereas male sexual coercion was associated with socially desirable components of narcissism. In congruence with previous research (Ryan et al., 2008; Southard, 2010), the gendered expressions found in this study may suggest that narcissistic behaviours are considered more socially acceptable and adaptive for males, whereas these behaviours (e.g. sexually dominant or instigating) may be conceptualised as beyond what is considered socially normative for women. Nevertheless, these findings are weakened by its complete reliance on the NPI as a measurement of narcissism.

Further adding to these limitations, some researchers exclude female participants entirely in their studies on the assumed basis that men generally possess higher levels of aggression and narcissism (e.g., Buck et al., 2014; Krusemark et al., 2018; Meier, 2004; Rinker, 2009; Talbot et al., 2015), and other researchers (e.g., Carton & Egan, 2017; Fields, 2012; Peterson & Dehart, 2014) fail to distinguish the gender of the perpetrator versus the victim. These characteristics and approaches within past research can be argued to perpetuate a failure to recognise gender identifications in the emergence of narcissistic personality attributes spanning its full expressions of grandiosity and vulnerability. Indeed, applying masculine derived concepts to females may not accurately capture narcissistic traits and the associated harmful impact females subject those close others. Over 15 years ago, Morf and Rhodewalt (2001) noted that research should “map out the forms of self-construction females employ, particularly when their selves are threatened” (page. 192). As demonstrated here, however, current theories of narcissism have still not attempted to explain how gender differences may emerge in this personality trait.

A recent study by  Green et al. (2020a) addressed these shortcomings in the literature by exploring gender differences in narcissism using a multidimensional assessment (i.e., the PNI; Pincus et al., 2009). This study found that females scored significantly higher on vulnerable narcissism than did males, and that vulnerable narcissism in females was the only significant predictor of physical/sexual and psychological abuse on a partner. This gender difference supported previous speculations suggesting that females may pursue their narcissistic goals in more discreet and indirect ways (e.g., Campbell & Miller, 2012; Morf & Rhodewalt, 2001). That is, whereas overt grandiosity in narcissistic males may create an acceptable norm about men being more exploitative and entitled, the same manifestation in females may be perceived as unconventional and thereby conceptualised as being beyond what is considered socially normative. These findings suggest that strategic attempts at self-construction may be markedly different, and gendered.

These expressions of behaviour are in congruence with a recent qualitative study on female narcissism as perpetrators of IPV ( Green et al., 2019). This study found that societal norms associated with gender roles shaped the motives and self-regulatory strategies in narcissistic females to obtain positions of power and control. These strategies were established through, for instance, adopting a ‘victim status’, playing the ‘mother card’, and exploiting legal and societal benefits to their advantage. If narcissism in females is likely influenced by lines of gender roles and feminine qualities, it is indeed conceivable that this destructive personality construct may go unnoticed in females due to their more hidden and vulnerable presentation of narcissism, given dominant measurements of grandiosity and longstanding focus on (narcissistic) males as perpetrators of IPV. These findings accentuate the need for future research to move beyond the masculine stereotype that is commonly conceptualised in theory and research, and linked to men’s perpetration of IPV.

## Existing gaps in theory and research

As this review has demonstrated, the nature and emergence of narcissism is most likely experienced differently in men and women, consequently resulting in particular implications for what has been traditionally understood and conceptualised as narcissism and for the related research which builds on these trait constellations (DSM/NPI). That is, depictions of NPD in the DSM-5 arguably contain criteria that entail and embody the male experience over that of the female. Therefore, the large body of research using the NPI is not only limited to overt grandiosity, but also limited to males. Another implications concerns the clinical utility of NPD, despite the evident trends in misdiagnoses and differential prevalence rates, the current literature still treats gender issues in narcissistic pathology as being separate from the criticisms commonly levelled at the criteria of this personality disorder.

Indeed, the criteria of NPD in the DSM-5 have been challenged on conceptual, clinical and empirical grounds, the most common criticism pertaining to the evident lack of narcissistic vulnerability (Cain et al., 2008; Dimaggio, 2012; Kealy & Rasmussen, 2012; Levy et al., 2011; Pincus & Lukowitsky, 2010; Reidy et al., 2008; Ronningstam, 2009). The failure to capture the phenomenology of NPD in its entirety has been said to most likely contribute to this disorder, exhibiting the lowest prevalence rate of the DSM personality disorders (Caligor et al., 2015; Miller et al., 2007; Russ et al., 2008). However, this is a finding which is inconsistent with the frequency of NPD diagnosis found in clinical practice (Cain et al., 2008; Euler et al., 2018), suggesting discrepancies exist between the diagnostic nomenclature as captured in the DSM-5 and the psychiatric phenomenon that is observed in clinical settings. It has been argued that changes in criteria are indicative of a concern to discriminate NPD from other pathologies, and in so doing, reducing comorbidity at the expense of the true phenomenological nature of NPD (Levy et al., 2011).

In terms of comorbidity, research has found that grandiose and vulnerable narcissism are associated with markedly different patterns of diagnostic overlap (Levy, 2012). Vulnerable narcissism has been associated with depression, anxiety, non-suicidal self-injury, suicide attempts (Miller & Campbell, 2008; Pincus & Lukowitsky, 2010; Russ et al., 2008; Thomas et al., 2012), BPD (Euler et al., 2018; Miller & Campbell, 2008; Miller et al., 2010; Pincus et al., 2009; Wright et al., 2010) and avoidant and dependent PD (Dickinson & Pincus, 2003; Miller et al., 2014). Grandiose narcissism, however, appears to more strongly correlate with antisocial personality disorder (ASPD; Stinson et al., 2008).

In fact, these differential patterns of comorbidity have also been shown as gender-specific: whereas men with narcissistic PD are more likely to be associated with antisocial PD and substance use disorders, women with narcissistic PD more frequently suffer from depressive and anxiety disorders and are more likely to have comorbid borderline PD (Stinson et al., 2008). Paris (2004) argued that differences in disorders may be explained by gender differences in traits (Costa et al., 2001; Ferguson & Eyre, 2000).These gender differences include males scoring higher on assertiveness and dominance which may, in turn, be reflective of a male predominance in externalising disorders (NPD, ASPD, substance abuse). Females, on the other hand, report higher levels of neuroticism, shame and nurturance, which may lead to a female predominance in internalising disorders (mood, anxiety, BPD; Paris, 2004).

Based on the above, it is important to acknowledge both the relative unawareness of understanding and approaching narcissistic pathology through the lens of gender, and how this unawareness has contributed to a poor clinical utility of NPD (e.g., low prevalence rates, diagnostic overlap, a lack of sufficient vulnerability). Instead, suggested proposals for improving the clinical utility and construct validity of NPD have been to revise the DSM criteria to include a number of specific features. First, to modify the current NPD criteria with explicit content covering vulnerable narcissistic features (Fossati et al., 2005; Miller et al., 2010; Pincus & Lukowitsky, 2010; Ronningstam, 2011), thereby indirectly addressing the gender issue. Second, to include narcissistic vulnerability as a specifier for NPD diagnoses (Miller et al., 2013). Third, to consider the ongoing debate of whether PDs in general, and pathological narcissism in particular, should be assessed using a dimensional trait-related rather than a categorical approach (Euler et al., 2018; Karterud et al., 2011).

This approach has been partially implemented in the DSM-5 with the aim to increase discriminant validity of PD diagnoses. This involves each PD to be diagnosed based on elevated scores of a specific number of traits from the dimensional trait model (i.e., negative affectivity, antagonism, detachment, psychoticism and disinhibition). It therefore uses a dimensional classification of personality pathology, rather than counting symptoms to inform a diagnosis. A final thought of revision concerns the construct validity of vulnerable narcissism, in light of the substantial degree of overlap with BPD and neuroticism (Miller et al., 2010, 2018). This is, specifically, whether it warrants its own place as a fully independent personality disorder construct rather than simply being a subtype of NPD, or if it is better suited as being a part of the BPD construct.

The existing literature is rife with ongoing debates regarding the descriptive characteristics of narcissism and diagnostic criteria that best exemplify the construct. These disparities have been poorly calibrated across the fields of psychiatry, clinical, and social/personality literature, reflecting enduring disagreement among clinicians and experts with regard to the central features of narcissism. For instance, research from the social/personality literature questions the notion that narcissistic grandiosity and vulnerability ‘co-exist’ (e.g., Miller et al., 2010, 2018), whereas the clinical literature suggests narcissistic individuals oscillate between the two dimensions (Cain et al., 2008; Ellison et al., 2013; Gore & Widiget, 2016; Pincus & Lukowitsky, 2010; Roberts & Huprich, 2012; Russ et al., 2008). More importantly, experts in the social/personality field generally believe that the grandiose features are more central to narcissism, whereas clinicians consider vulnerability to be more central (Ackerman et al., 2017).

This definitional ambiguity is reflected in the diversity of measurements available to assess narcissism; a state of affairs which has resulted in difficulties to integrate the literature as various ‘camps’ define the construct differently (Pincus & Lukowitsky, 2010). At this juncture, the generality of findings are limited to, and dependent on, the theoretical assumptions about the construct. For instance, the PNI (Pincus et al., 2009) was developed to measure pathological narcissism as it is conceptualised in clinical theory. However, the PNI has been criticised for emphasising vulnerable traits, thereby deviating from conceptions of NPD in the DSM and the related research using the NPI which are, instead, over-reliant on grandiose features. The theoretical definitions of narcissism tend to therefore emphasise either one of its polarities. The field’s fractured state allows for this diversity, further highlighting the need for a solution that unites these sub-disciplines and precision in definition, whilst at the same time appreciates the gender issues involved. The following section makes the case for a theoretical re-synthesis of narcissism that aims to facilitate integration across the subfields inclusive of gender contributions.

## Moving forward with a literature of narcissism inclusive of gender issues

A stronger theoretical foundation for the conceptualisation of narcissism may be that derived from the perspective of a Five Factor Model (FFM; Widiger & Costa, 2013), which consists of the following broad domains: neuroticism, extraversion versus introversion, openness, agreeableness versus antagonism, and conscientiousness. Such a framework follows the considerable body of research supporting the contention that personality disorders are the severe form of personality traits, and thereby better conceptualised as a five-domain dimensional trait model. The dimensional trait model has also been rated as more effective for clinical purposes (Bernstein et al., 2007; Hansen, 2019; Morey et al., 2011), compared to the existing categorical approach in the DSM-5. The FFNI model represents an extension of the FFM and specifically encompasses the more extreme and maladaptive personality facets. Based on this literature, the FFNI (Miller et al., 2013) was developed relatively recently to complement other multidimensional assessments (i.e. the PNI) in assessing the grandiose and vulnerable features of narcissism. The theoretical and empirical underpinnings of the FFNI differs from the PNI, however, in that the former is based on the large empirical literature of assessments of pathological personality traits from an FFM perspective (see Glover et al., 2012; Miller et al., 2013, 2014).

Another advantage of conceptualising narcissism from an FFM perspective is that the gender differences of FFM traits are well-studied, such that females consistently report higher neuroticism and males score higher on antagonism (Costa et al., 2001; Ferguson & Eyre, 2000; Paris, 2004). These differences also resemble the differential prevalence rates among males and females in the DSM PDs (see Lynam & Widiger, 2007). Although a recent study by Suzuki et al. (2019) found that the dimensional trait model in the DSM is structurally equivalent across males and females, females were found to have higher scores on Negative Affectivity, whereas males had higher scores on Detachment, Antagonism, Disinhibition and Psychoticism when examined at the latent trait mean levels. It is both suggested and also strongly emphasised here that differences in narcissistic pathology may be rooted in trait dimensions shaped by gender. For this reason, gender may play a key factor that partly determines specific psychopathological constellations.

Moving forward, the FFNI approach offers a potential advance in the conceptualisation of narcissism. On the one hand, this would allow the field to unify the empirically and clinically derived concepts about the construct. On the other hand, such a framework can pinpoint gender-specific expressions in the presentation of narcissistic personality attributes, thereby constituting an important step towards a conceptual model inclusive of gender factors in these manifestations.

In addition to employing a more gender-sensitive assessment of narcissism, future research should consider examining sex differences in narcissism. For instance, narcissism in males has been associated with heightened cortisol reactivity to psychosocial stressors (Edelstein et al., 2010) and higher cortisol levels than in narcissistic females (Reinhard et al., 2012). Given the importance of genetic differences between men and women, which may partly shape the effects of gender on personality (Paris, 2004), future research should consider the role of biological sex differences in males and females with narcissistic pathology. Such foci may help refine both psychosocial and biological approaches to treatment. Another line of enquiry for future research could involve the investigation of sex differences in narcissism through other models, since the bulk of research that conceptualises narcissism is based on a trait approach (Campbell & Miller, 2011). Future research could therefore explore sex differences in narcissism from, for instance, an evolutionary perspective as such an approach may provide useful insights that are not offered by prominent theories discussed in this review.

Indeed, given the nontrivial heritability of narcissism (Vernon et al., 2008), manifestations of narcissism could be partially shaped by evolutionary processes, such as short-term mating (STM), coercive sexual tendencies and attractiveness (for reviews, see Holtzman & Donnellan, 2015; Holtzman & Strube, 2012). Holtzman and Strube (2012) argue that males are more likely than females to seek the more reproductive benefits of STM, and as a result, coercive tendencies commonly apply more to men than women. It may be speculated that males, when threatened, may resort to evolutionarily well-established strategies of power, dominance, inflated self-image and externalising behaviour. Females, on the other hand, may resort to other well-established evolutionary strategies, such as attaining attention through promoting a self-image that signals sexuality, beauty and attractiveness. Future research could explore these speculations, as an evolutionary approach can complement the literature and enhance theoretical understanding regarding gender differences in narcissistic presentation.

It is also recommended for future research to undertake more longitudinal designs in order to better understand the development and features of narcissism and how they relate to gender over time. For instance, longitudinal, genetically informed designs can further identify aetiological factors (parent-child interactions) associated with grandiose and vulnerable narcissism (Luo & Cai, 2018), with a focus on gender differences. Ecological momentary assessment (EMA) can also be employed to assess momentary periods within a narcissistic individual’s life over time (Wright, 2014). Future research could focus on how grandiose and vulnerable features fluctuate over time within males and females with both subclinical and clinical degrees of narcissism. Lastly, since gender constructs continually change, and socially accepted gender roles differ greatly across cultures, so do the manifestations of narcissism (e.g., Campbell & Miller, 2012; Foster et al., 2003). Future studies could there investigate the influence of cultural differences in the manifestations of narcissism in males and females, within more diverse cultures. A particular focus could be to compare how the characteristics of narcissism vary by gender in more collectivist societies, as these societies place a greater focus on others compared to more western individualistic societies where narcissism is arguably higher due to promotion of self- focus (Foster et al., 2003). Through such an undertaking, a more complex picture and rich analysis of the ways in which gender and narcissism interact and influence each other will arguably emerge.

## Conclusion

Although extensive, the research on narcissism across clinical theory and empirical research (DSM/NPI) is characterised by a relative ignorance regarding how gender disparities manifest in narcissistic expression, behaviour and functioning. Notably, the overrepresentation of males and the concomitant underrepresentation of females in the literature is indicative of the symptomatology of the narcissistic personality (NPI/DSM), which closely resembles the masculine stereotype of males in the society. This review has shown that, whilst the core of narcissism operates similar across gender, the outward expressions tend to differ. It is *echoed* here that failure to appreciate salient gender differences in the expression of narcissism will likely result in enormous negative consequences across diagnostic assessment, treatment, gender-appropriate interventions for offending behaviours, and the necessary theoretical knowledge to inform these identified areas of concern. To further advance the field, it is strongly recommended that future research employ a multifaceted assessment of narcissism, such as the FFNI, to further enrich theoretical understanding of the extent to which articulations of Narcissus and Echo have been gender informed. In so doing, the field can move towards a more robust and integrated literature on narcissism that is inclusive of gender issues.

## References

[bibr524-00332941211027322] Ackerman R. A. Hands A. J. Donnellan M. B. Hopwood C. J. Witt E. A. (2017). Experts’ Views Regarding the Conceptualization of Narcissism. Journal of Personality Disorders, 31, 3, 346–361. 10.1521/pedi_2016_30_254 27322575

[bibr219-00332941211027322] Adler G. (1986). Psychotherapy of the narcissistic personality disorder patient: two contrasting approaches. American Journal of Psychiatry, 143, 430–436. 3513633 10.1176/ajp.143.4.430

[bibr310-00332941211027322] Akhtar S. Thomson J. A. (1982). Overview: Narcissistic personality disorder. The American Journal of Psychiatry, 139(1), 12–20. 7034551 10.1176/ajp.139.1.12

[bibr1-00332941211027322] American Psychiatric Association. (2013). *Diagnostic and statistical manual of mental disorders: DSM-5*.

[bibr2-00332941211027322] Anderson K. G. Sankis L. M. Widiger T. A. (2001). Pathology versus statistical infrequency: Potential sources of gender bias in personality disorder criteria. The Journal of Nervous and Mental Disease, 189(10), 661–668. 11708666 10.1097/00005053-200110000-00002

[bibr211-00332941211027322] Barnett M. D. Powell H. A. (2016). Self-esteem mediates narcissism and aggression among women, but not men: A comparison of two theoretical models of narcissism among college students. Personality and Individual Differences, 89, 100–104.

[bibr212-00332941211027322] Barnett M. D. Sharp K. J. (2017). Narcissism, gender, and evolutionary theory: The role of private and public self-absorption. Personality and Individual Differences, 104, 326–332.

[bibr3-00332941211027322] Bernstein D. P. Iscan C. Maser J. (2007). Opinions of personality disorder experts regarding the DSM-IV personality disorders classification system. Journal of Personality Disorders, 21(5), 536–551. 17953505 10.1521/pedi.2007.21.5.536

[bibr213-00332941211027322] Besser A. Priel B. (2009). Emotional responses to a romantic partner's imaginary rejection: The roles of attachment anxiety, covert narcissism, and self-evaluation. Journal of Personality, 77(1), 287–325. 19076997 10.1111/j.1467-6494.2008.00546.x

[bibr4-00332941211027322] Blinkhorn V. Lyons M. Almond L. (2015). The ultimate femme fatale? Narcissism predicts serious and aggressive sexually coercive behaviour in females. Personality and Individual Differences, 87, 219–223.

[bibr5-00332941211027322] Blinkhorn V. Lyons M. Almond L. (2019). Criminal minds: Narcissism predicts offending behavior in a Non-Forensic sample. Deviant Behavior, 40(3), 353–360.

[bibr6-00332941211027322] Braamhorst W. Lobbestael J. Emons W. H. M. Arntz A. Witteman C. L. M. Bekker M. H. J. (2015). Sex bias in classifying borderline and narcissistic personality disorder. Journal of Nervous & Mental Disease, 203(10), 804–808. 26421970 10.1097/NMD.0000000000000371

[bibr8-00332941211027322] Buck N. M. L. Leenaars P. E. M. Emmelkamp P. M. G. van M. H. J. C. (2014). Personality traits are related to intimate partner violence among securely attached individuals. Journal of Family Violence, 29(3), 235–246.

[bibr9-00332941211027322] Cain N. M. Pincus A. L. Ansell E. B. (2008). Narcissism at the crossroads: Phenotypic description of pathological narcissism across clinical theory, social/personality psychology, and psychiatric diagnosis. Clinical Psychology Review, 28(4), 638–656. 18029072 10.1016/j.cpr.2007.09.006

[bibr10-00332941211027322] Caiozzo C. N. Houston J. Grych J. (2016). Predicting aggression in late adolescent romantic relationships: A short-term longitudinal study. Journal of Adolescence, 53, 237–248. 27816698 10.1016/j.adolescence.2016.10.012

[bibr11-00332941211027322] Caligor E. Levy K. N. Yeomans F. E. (2015). Narcissistic personality disorder: Diagnostic and clinical challenges. American Journal of Psychiatry, 172(5), 415–422. 25930131 10.1176/appi.ajp.2014.14060723

[bibr1211-00332941211027322] Campbell, W. K., & Miller, J. D. (2011). *The handbook of narcissism and narcissistic personality disorder: Theoretical approaches, empirical findings, and treatments*. Hoboken, N.J: John Wiley.

[bibr12-00332941211027322] Campbell W. K. Miller J. D. (2012). The handbook of narcissism and narcissistic personality disorder: Theoretical approaches, empirical findings, and treatments. John Wiley.

[bibr13-00332941211027322] Capron E. W. (2004). Types of pampering and the narcissistic personality trait. Journal of Individual Psychology, 60, 76–93.

[bibr15-00332941211027322] Carroll L. (1989). A comparative study of narcissism, gender, and sex-role orientation among bodybuilders, athletes, and psychology students. Psychological Reports, 64(3), 999–1006.

[bibr16-00332941211027322] Carton H. Egan V. (2017). The dark triad and intimate partner violence. Personality and Individual Differences, 105, 84–88.

[bibr18-00332941211027322] Corry N. Merritt R. D. Mrug S. Pamp B. (2008). The factor structure of the narcissistic personality inventory. Journal of Personality Assessment, 90(6), 593–600. 18925501 10.1080/00223890802388590

[bibr19-00332941211027322] Costa P. T. Terracciano A. McCrae R. R. (2001). Gender differences in personality traits across cultures: Robust and surprising findings. Journal of Personality and Social Psychology, 81(2), 322–331. 11519935 10.1037/0022-3514.81.2.322

[bibr21-00332941211027322] Cramer P. (2015). Adolescent parenting, identification, and maladaptive narcissism. Psychoanalytic Psychology, 32(4), 559–579.

[bibr22-00332941211027322] Dickinson K. A. Pincus A. L. (2003). Interpersonal analysis of grandiose and vulnerable narcissism. Journal of Personality Disorders, 7(3), 188–207. 10.1521/pedi.17.3.188.22146 12839099

[bibr312-00332941211027322] Dimaggio G. (2012). Narcissistic personality disorder: Rethinking what we know. Psychiatric Times, 29(7), 17–25.

[bibr330-00332941211027322] Edelstein R. S. Yim I. S. Quas J. A. (2010). Narcissism predicts heightened cortisol reactivity to a psychosocial stressor in men. Journal of Research in Personality, 44, 5, 565–572. 10.1016/j.jrp.2010.06.008 PMC2976540 21076653

[bibr215-00332941211027322] Ellis H. (1898). Auto-erotism: A psychological study. Alienist and Neurologist, 19, 260–299.

[bibr23-00332941211027322] Ellison W. D. Levy K. N. Cain N. M. Ansell E. B. Pincus A. L. (2013). The impact of pathological narcissism on psychotherapy utilization, initial symptom severity, and early-treatment symptom change: A naturalistic investigation. Journal of Personality Assessment, 95(3), 291–300. 23186259 10.1080/00223891.2012.742904

[bibr24-00332941211027322] Euler S. Stöbi D. Sowislo J. Ritzler F. Huber C. G. Lang U. E. Wrege J. Walter M. (2018). Grandiose and vulnerable narcissism in borderline personality disorder. Psychopathology, 51(2), 110–121. 29466803 10.1159/000486601

[bibr25-00332941211027322] Ferguson T. J. Eyre H. L. (2000). Engendering gender differences in shame and guilt: Stereotypes, socialization, and situational pressures. In Fischer A. H. (Ed.), Gender and emotion: Social psychological perspectives (pp. 254–276). Cambridge University Press.

[bibr26-00332941211027322] Fields S. K. (2012). *Narcissism and intimate partner violence: An establishment of the link and investigation of multiple potential mediators*. *Digital Commons.* East Tennessee State University.

[bibr313-00332941211027322] Filippini S. (2005). Perverse relationships: The perspective of the perpetrator. The International Journal of Psychoanalysis. 86, 755–773. 16096074 10.1516/gku9-etye-qe00-yexy

[bibr435-00332941211027322] Foster J. D. Campbell W. K. Twenge J. M. (2003). Individual differences in narcissism: Inflated self-views across the lifespan and around the world. Journal of Research in Personality, 37(6), 469–486.

[bibr27-00332941211027322] Fossati A. Beauchaine T. P. Grazioli F. Carretta I. Cortinovis F. Maffei C. (2005). A latent structure analysis of diagnostic and statistical manual of mental disorders, fourth edition, narcissistic personality disorder criteria. Comprehensive Psychiatry, 46(5), 361–367. 16122536 10.1016/j.comppsych.2004.11.006

[bibr28-00332941211027322] Freud S. (1914/1957). On narcissism: An introduction. In Strachey J. (Ed. and Trans.), The standard edition of the complete psychological works of Sigmund Freud (pp. 67–104). Hogarth Press.

[bibr319-00332941211027322] Girgis A. (2006). Violence from self-Love: narcissism and aggression in the face of ego threat. Psychology Honors Theses, 3.

[bibr2988-00332941211027322] Glover N. Miller J. D. Lynam D. R. Crego C. Widiger T. A. (2012). The Five-Factor narcissism inventory: A Five-Factor measure of narcissistic personality traits. Journal of Personality Assessment, 94(5), 500–512. 22475323 10.1080/00223891.2012.670680

[bibr30-00332941211027322] Gormley B. Lopez F. G. (2010). Correlates of psychological abuse perpetration in college dating relationships. Journal of College Counseling, 13(1), 4–16. 10.1177/0886260509334404 19520968

[bibr325-00332941211027322] Gore W. L. Widiger T. A. (2016). Fluctuation between grandiose and vulnerable narcissism. Personality Disorders, 7, 4, 363–371. 26986960 10.1037/per0000181

[bibr97-00332941211027322] Green A. Charles K. (2019). Voicing the victims of narcissistic partners: A qualitative analysis of responses to narcissistic injury and Self-Esteem regulation. Sage Open, 9(2), 215824401984669–215824401984610.

[bibr98-00332941211027322] Green A. Charles K. MacLean R. (2019). Perceptions of female narcissism in intimate partner violence: A thematic analysis. *Qualitative Methods in Psychology Bulletin*, 28, 13–27.

[bibr99-00332941211027322] Green A. MacLean R. Charles K. (2020a). Recollections of parenting styles in the development of narcissism: The role of gender. Personality and Individual Differences, 167, 110246.

[bibr100-00332941211027322] Green A. MacLean R. Charles K. (2020b). Unmasking gender differences in narcissism within intimate partner violence. Personality and Individual Differences, 167, 110247.

[bibr31-00332941211027322] Grijalva E. Newman D. A. Tay L. Donnellan M. B. Harms P. D. Robins R. W. Yan T. (2015). Gender differences in narcissism: A meta-analytic review. Psychological Bulletin, 141(2), 261–310. 25546498 10.1037/a0038231

[bibr32-00332941211027322] Grilo C. M. Becker D. F. Fehon D. C. Walker M. L. Edell W. S. McGlashan T. H. (1996). Gender differences in personality disorders in psychiatrically hospitalized adolescents. The American Journal of Psychiatry, 153(8), 1089–1091. 8678180 10.1176/ajp.153.8.1089

[bibr323-00332941211027322] Gunderson J.G. Ronningstam E. (2001). Differentiating narcissistic and antisocial personality disorders. Journal of Personality Disorders, 15(2), 103–109. 11345846 10.1521/pedi.15.2.103.19213

[bibr33-00332941211027322] Haaken J. (1983). Sex differences and narcissistic disorders. American Journal of Psychoanalysis, 43, 315–324. 6666778 10.1007/BF01248460

[bibr328-00332941211027322] Hansen S. J. Christensen S. Kongerslev M. T. First M. B. Widiger T. A. Simonsen E. Bach B. (2019). Mental health professionals' perceived clinical 225 utility of the ICD-10 vs. ICD-11 classification of personality disorders. Personality and Mental Health, 13, 2, 84–95. 30989832 10.1002/pmh.1442

[bibr34-00332941211027322] Hoertel N. Lavaud P. Guerin-Langlois C. Rene M. Schuster J.-P. Lemogne C. Limosin F. Schuster J.-P. (2018). Examining sex differences in DSM-IV-TR narcissistic personality disorder symptom expression using item response theory (IRT). Psychiatry Research, 260, 500–507. 29291575 10.1016/j.psychres.2017.12.031 PMC6002876

[bibr35-00332941211027322] Holtzman N. S. Strube M. J. (2012). The intertwined evolution of narcissism and Short-Term mating: An emerging hypothesis. In Campbell W. K. Miller J. D. (Eds.), The handbook of narcissism and narcissistic personality disorders. Wiley. 210–220

[bibr432-00332941211027322] Holtzman N. S. Donnellan M. B. (2015). The roots of Narcissus: Old and new models of the evolution of narcissism. In Zeigler-Hill V. Welling L. L. M. Shackelford T. K. (Eds.), Evolutionary perspectives on social psychology (pp. 479–489). New York: Springer.

[bibr36-00332941211027322] Horton R. S. Bleau G. Drwecki B. (2006). Parenting narcissus: What are the links between parenting and narcissism? Journal of Personality, 74(2), 345–376. 16529580 10.1111/j.1467-6494.2005.00378.x

[bibr37-00332941211027322] Huxley E. Bizumic B. (2017). Parental invalidation and the development of narcissism. The Journal of Psychology, 151(2), 130–147. 27858527 10.1080/00223980.2016.1248807

[bibr38-00332941211027322] Jane J. S. Oltmanns T. F. South S. C. Turkheimer E. (2007). Personality pathology—Gender bias in diagnostic criteria for personality disorders: An item response theory analysis. Journal of Abnormal Psychology, 116(1), 166–175. 17324027 10.1037/0021-843X.116.1.166 PMC4372614

[bibr39-00332941211027322] Jonason P. K. Davis M. D. (2018). A gender role view of the dark triad traits. Personality and Individual Differences, 125, 102–105.

[bibr40-00332941211027322] Karterud S. Øien M. Pedersen G. (2011). Validity aspects of the diagnostic and statistical manual of mental disorders, fourth edition, narcissistic personality disorder construct. Comprehensive Psychiatry, 52(5), 517–526. 21193181 10.1016/j.comppsych.2010.11.001

[bibr41-00332941211027322] Kealy D. Ogrodniczuk J. S. (2011). Narcissistic interpersonal problems in clinical practice. Harvard Review of Psychiatry, 19(6), 290–301. 22098325 10.3109/10673229.2011.632604

[bibr42-00332941211027322] Kealy D. Rasmussen B. (2012). Veiled and vulnerable: The other side of grandiose narcissism. Clinical Social Work Journal, 40(3), 356–365.

[bibr44-00332941211027322] Kernberg O. (1975). Borderline conditions and pathological narcissism. Jason Aronson.

[bibr45-00332941211027322] Klonsky E. D. Jane J. S. Turkheimer E. Oltmanns T. F. (2002). Gender role and personality disorders. Journal of Personality Disorders, 16(5), 464–476. 12489312 10.1521/pedi.16.5.464.22121 PMC4364134

[bibr46-00332941211027322] Kohut H. (1977). The restoration of the self. International Universities Press.

[bibr47-00332941211027322] Krusemark E. A. Campbell W. K. Crowe M. L. Miller J. D. (2018). Comparing self-report measures of grandiose narcissism, vulnerable narcissism, and narcissistic personality disorder in a male offender sample. Psychological Assessment, 30(7), 984–990. 29792507 10.1037/pas0000579

[bibr48-00332941211027322] Lamkin J. Lavner J. A. Shaffer A. (2017). Narcissism and observed communication in couples. Personality and Individual Differences, 105, 224–228.

[bibr49-00332941211027322] Lavner J. A. Lamkin J. Miller J. D. Campbell W. K. Karney B. R. (2016). Narcissism and newlywed marriage: Partner characteristics and marital trajectories. Personality Disorders: Theory, Research, and Treatment, 7(2), 169–179. 10.1037/per0000137 PMC4688247 26098378

[bibr50-00332941211027322] Levy K. N. (2012). Subtypes, dimensions, levels, and mental states in narcissism and narcissistic personality disorder. Journal of Clinical Psychology, 68(8), 886–897. 22740389 10.1002/jclp.21893

[bibr51-00332941211027322] Levy K. N. Ellison W. D. Reynoso J. S. (2011). A historical review of narcissism and narcissistic personality. In Campbell W. K. Miller J. D. (Eds.). Handbook of narcissism and narcissistic personality disorder: Theoretical approaches, empirical findings, and treatments (pp. 3–12). Wiley.

[bibr52-00332941211027322] Lindsay K. A. Sankis L. M. Widiger T. A. (2000). Gender bias in self-report personality disorder inventories. Journal of Personality Disorders, 14(3), 218–232. 11019746 10.1521/pedi.2000.14.3.218

[bibr318-00332941211027322] Logan C. (2009). Narcissism. In McMurran, M., & Howard, R. C. (2009). Personality, personality disorder, and violence. New York, Wiley.

[bibr53-00332941211027322] Lukowitsky M. R. Pincus A. L. (2013). Interpersonal perception of pathological narcissism: A social relations analysis. Journal of Personality Assessment, 95(3), 261–273. 23406324 10.1080/00223891.2013.765881

[bibr433-00332941211027322] Luo Y.L.L. Cai H. (2018). The Etiology of Narcissism: A Review of Behavioral Genetic Studies. In: Hermann A. Brunell A. Foster J. (eds) Handbook of Trait Narcissism. Springer, Cham. 149–156.

[bibr54-00332941211027322] Lynam D. R. Widiger T. A. (2007). Using a general model of personality to understand sex differences in the personality disorders. Journal of Personality Disorders, 21(6), 583–602. 18072861 10.1521/pedi.2007.21.6.583

[bibr55-00332941211027322] Lyons M. Morgan K. Thomas J. Hashmi A. A. (2013). Patterns of parental warmth, attachment, and narcissism in young women in United Arab Emirates and the United Kingdom. Individual Differences Research, 11(4), 149–158.

[bibr56-00332941211027322] Mechanic K. L. Barry C. T. (2015). Adolescent grandiose and vulnerable narcissism: Associations with perceived parenting practices. Journal of Child and Family Studies, 24(5), 1510–1518.

[bibr57-00332941211027322] Meier M. J. (2004). Exploring narcissism in a group of male batterers. Dissertation Abstracts International, 64(10-A), 3846–3846.

[bibr58-00332941211027322] Miller J. D. Campbell W. K. (2008). Comparing clinical and social-personality conceptualizations of narcissism. Journal of Personality, 76, 449–476. 18399956 10.1111/j.1467-6494.2008.00492.x

[bibr59-00332941211027322] Miller J. D. Campbell W. K. Pilkonis P. A. (2007). Narcissistic personality disorder: Relations with distress and functional impairment. Comprehensive Psychiatry, 48(2), 170–177. 17292708 10.1016/j.comppsych.2006.10.003 PMC1857317

[bibr60-00332941211027322] Miller J. D. Dir A. Gentile B. Wilson L. Pryor L. R. Campbell W. K. (2010). Searching for a vulnerable dark triad: Comparing factor 2 psychopathy, vulnerable narcissism, and borderline personality disorder. Journal of Personality, 78(5), 1529–1564. 20663024 10.1111/j.1467-6494.2010.00660.x

[bibr61-00332941211027322] Miller J. D. Gentile B. Wilson L. Campbell W. K. (2013). Grandiose and vulnerable narcissism and the DSM-5 pathological personality trait model. Journal of Personality Assessment, 95(3), 284–290. 22594764 10.1080/00223891.2012.685907

[bibr63-00332941211027322] Miller J. D. Lynam D. R. Vize C. Crowe M. Sleep C. Maples-Keller J. L. Few L. R. Campbell W. K. (2018). Vulnerable narcissism is (mostly) a disorder of neuroticism. Journal of Personality, 86(2), 186–199. 28170100 10.1111/jopy.12303

[bibr64-00332941211027322] Miller J. D. McCain J. Lynam D. R. Few L. R. Gentile B. MacKillop J. Campbell W. K. (2014). A comparison of the criterion validity of popular measures of narcissism and narcissistic personality disorder via the use of expert ratings. Psychological Assessment, 26(3), 958–969. 24773036 10.1037/a0036613

[bibr65-00332941211027322] Millon T. (1981). Disorders of personality: DSM III: Axis II. John Wiley.

[bibr29-00332941211027322] Morey L. C. Berghuis H. Bender D. S. Verheul R. Krueger R. F. Skodol A. E. (2011). Toward a Model for Assessing Level of Personality Functioning in DSM–5, Part II: Empirical Articulation of a Core Dimension of Personality Pathology. Journal of Personality Assessment, 93, 4, 347–353. 22804673 10.1080/00223891.2011.577853

[bibr66-00332941211027322] Morf C. C. Rhodewalt F. (2001). Unraveling the paradoxes of narcissism: A dynamic self-regulatory processing model. Psychological Inquiry, 12(4), 177–196.

[bibr67-00332941211027322] O’leary J. Wright F. (1986). Shame and gender issues in pathological narcissism. Psychoanalytic Psychology, 3(4), 327–339.

[bibr68-00332941211027322] Onofrei L. (2009). A critical examination of the theoretical and empirical overlap between overt narcissism and male narcissism and between covert narcissism and female narcissism. *Theses, Dissertations, and Projects*, 11–33.

[bibr69-00332941211027322] Paris J. (2004). Gender differences in personality traits and disorders. Current Psychiatry Reports, 6, 71–74. 14738709 10.1007/s11920-004-0042-8

[bibr70-00332941211027322] Paris J. Chenard-Poirier M.-P. Biskin R. (2013). Antisocial and borderline personality disorders revisited. Comprehensive Psychiatry, 54 (4), 321–325. 23200574 10.1016/j.comppsych.2012.10.006

[bibr316-00332941211027322] Perry J. D. Perry J. C. (2004). Conflicts, defenses and the stability of narcissistic personality features. Psychiatry, 67(4), 310–30. 15801375 10.1521/psyc.67.4.310.56570

[bibr71-00332941211027322] Peterson J. L. DeHart T. (2014). In defense of self-love: An observational study on narcissists' negative behavior during romantic relationship conflict. Self and Identity, 13(4), 477–490.

[bibr72-00332941211027322] Philipson I. (1985). Gender and narcissism. Psychology of Women Quarterly, 9(2), 213–228.

[bibr73-00332941211027322] Pincus A. L. Pimentel C. A. Cain N. M. Wright A. G. C. Levy K. N. Ansell E. B. (2009). Initial construction and validation of the pathological narcissism inventory. Psychological Assessment, 21(3), 365–379. 19719348 10.1037/a0016530

[bibr74-00332941211027322] Pincus A. L. Wright A. G. C. Cain N. M. (2014). Narcissistic grandiosity and narcissistic vulnerability in psychotherapy. Personality Disorders: Theory, Research, and Treatment, 5(4), 439–443. 10.1037/per0000031 24446581

[bibr326-00332941211027322] Pincus A. L. Lukowitsky M. R. (2010). Pathological Narcissism and Narcissistic Personality Disorder. Annual Review of Clinical Psychology, 6(1), 421–446. 10.1146/annurev.clinpsy.121208.131215 20001728

[bibr327-00332941211027322] Pincus A. L. Pimentel C. A. Cain N. M. Wright A. G. C. Levy K. N. Ansell E. B. (2009). Initial Construction and Validation of the Pathological Narcissism Inventory. Psychological Assessment, 21(3), 365–379. 19719348 10.1037/a0016530

[bibr75-00332941211027322] Pulay A. J. Goldstein R. B. Grant B. F. (2012). *Sociodemographic correlates of DSM-IV narcissistic personality disorder: Results from the wave 2 National Epidemiologic Survey on Alcohol and Related Conditions (NESARC)*. In W. K. Campbell & J. D. Miller (Eds.), Handbook of narcissism and narcissistic personality disorder: Theoretical approaches, empirical findings, and treatments (pp. 167–180). Hoboken, NJ: Wiley.

[bibr76-00332941211027322] Pullen A. Rhodes C. (2008). It’s all about me!’: Gendered narcissism and leaders’ identity work. Leadership, 4(1), 5–25.

[bibr216-00332941211027322] Rank O. (1911). A contribution to narcissism. Jahrbush fur Psychoanalytische und Psychopathologische Forschungen, 3, 401–426.

[bibr314-00332941211027322] Raskin R. Terry H. (1988). A principal-components analysis of the Narcissistic Personality Inventory and further evidence of its construct validity. Journal of Personality and Social Psychology, 54, 890–902. 3379585 10.1037//0022-3514.54.5.890

[bibr218-00332941211027322] Reich W. (1933/1949). Character analysis. T.P. Wolfe (Trans.; 3rd Edition 1949, original work published 1933). New York: Orgone Institute Press.

[bibr77-00332941211027322] Reidy D. E. Zeichner A. Foster J. D. Martinez M. A. (2008). Effects of narcissistic entitlement and exploitativeness on human physical aggression. Personality and Individual Differences, 44(4), 865–875.

[bibr331-00332941211027322] Reinhard D. A. Konrath S. H. Lopez W. D. Cameron H. G. (2012). Expensive egos: narcissistic males have higher cortisol. Plos One, 7, 1–8. 10.1371/journal.pone.0030858 PMC3264640 22292062

[bibr78-00332941211027322] Richman J. A. Flaherty J. A. (1988). “Tragic man” and “tragic woman”: Gender differences in narcissistic styles. Psychiatry (Psychiatry), 51(4), 368–377. 3237899 10.1080/00332747.1988.11024413

[bibr79-00332941211027322] Rinker L. S. (2009). Narcissism and type of violent relationships for perpetrators of intimate partner violence. Dissertation Abstracts International, 70, 5802.

[bibr315-00332941211027322] Roberts C. R. D. Huprich S. K. (2012). Categorical and Dimensional Models of Pathological Narcissism: The Case of Mr. Jameson. Journal of Clinical Psychology, 68, 8, 898–907. 10.1002/jclp.21894 22730014

[bibr317-00332941211027322] Robinson H. Graham F. V. (2004). Understanding Narcissism in Clinical Practice (Society of Analytical Psychology Monograph Series). Karnac Books.

[bibr80-00332941211027322] Rohmann E. Neumann E. Herner M. J. Bierhoff H.-W. (2012). Grandiose and vulnerable narcissism: Self-construal, attachment, and love in romantic relationships. European Psychologist, 17(4), 279–290.

[bibr81-00332941211027322] Ronningstam E. (2009). Narcissistic personality disorder: Facing DSM-V. Psychiatry Annals, 39, 111–121.

[bibr82-00332941211027322] Ronningstam E. (2006). Identifying and understanding the narcissistic personality. Oxford University Press.

[bibr83-00332941211027322] Ronningstam E. (2011). Narcissistic personality disorder: A clinical perspective. Journal of Psychiatric Practice, 17, 89–99. 21430487 10.1097/01.pra.0000396060.67150.40

[bibr84-00332941211027322] Russ E. Shedler J. Bradley R. Westen D. (2008). Refining the construct of narcissistic personality disorder: Diagnostic criteria and subtypes. American Journal of Psychiatry, 165(11), 1473–1481. 18708489 10.1176/appi.ajp.2008.07030376

[bibr85-00332941211027322] Ryan K. M. Weikel K. Sprechini G. (2008). Gender differences in narcissism and courtship violence in dating couples. Sex Roles, 58(11–12), 802–813.

[bibr86-00332941211027322] Samuels J. Eaton W. W. Bienvenu O. J. Brown C. H. Costa P. T. Nestadt G. (2002). Prevalence and correlates of personality disorders in a community sample. British Journal of Psychiatry, 180(06), 536–542. 10.1192/bjp.180.6.536 12042233

[bibr87-00332941211027322] Schulte H. B. Habel U. (2018). Sex differences in personality disorders. Current Psychiatry Reports, 20(12), 1–7. 30306417 10.1007/s11920-018-0975-y

[bibr214-00332941211027322] Sherry S. B. Gralnick T. M. Hewitt P. L. Sherry D. L. Flett G. L. (2014). Perfectionism and narcissism: Testing unique relationships and gender differences. Personality and Individual Differences, 61, 52–56

[bibr88-00332941211027322] Simmons C. A. Lehmann P. Cobb N. Fowler C. R. (2005). Personality profiles of women and men arrested for domestic violence: An analysis of similarities and differences. Journal of Offender Rehabilitation, 41(4), 63–82.

[bibr89-00332941211027322] Skodol A. E. Bender D. S. (2003). Why are women diagnosed borderline more than men? Psychiatric Quarterly, 74(4), 349–360. 14686459 10.1023/a:1026087410516

[bibr90-00332941211027322] Southard A. C. (2010). Sex differences in narcissism: Expression of and relationships with the exploitativeness/entitlement factor. Western Carolina University.

[bibr520-00332941211027322] Sprecher S. Brooks J. E. Avogo W. (2013). Self-Esteem Among Young Adults: Differences and Similarities Based on Gender, Race, and Cohort (1990–2012). Sex Roles, 69, 264–275.

[bibr91-00332941211027322] Stinson F. S. Dawson D. A. Goldstein R. B. Chou S. P. Huang B. Smith S. M. Ruan W. J. Grant B. F. (2008). Prevalence, correlates, disability, and comorbidity of DSM-IV narcissistic personality disorder: Results from the wave 2 national epidemiologic survey on alcohol and related conditions. The Journal of Clinical Psychiatry, 69(7), 1033–1045. 18557663 10.4088/jcp.v69n0701 PMC2669224

[bibr92-00332941211027322] Suzuki T. South S. C. Samuel D. B. Wright A. G. C. Yalch M. M. Hopwood C. J. Thomas K. M. (2019). Measurement invariance of the DSM–5 section III pathological personality trait model across sex. Personality Disorders: Theory, Research, and Treatment, 10(2), 114–122. 10.1037/per0000291 PMC6310673 29952589

[bibr93-00332941211027322] Talbot F. Babineau M. Bergheul S. (2015). Narcissism and self-esteem dimensions as predictors of aggression in intimate partner violence. Annales Medico-Psychologiques, 173(2), 193–196.

[bibr94-00332941211027322] Thomas K. M. Wright A. G. C. Lukowitsky M. R. Donnellan M. B. Hopwood C. J. (2012). Evidence for the criterion validity and clinical utility of the pathological narcissism inventory. Assessment, 19(2), 135–145. 22315481 10.1177/1073191112436664 PMC3354008

[bibr95-00332941211027322] Torgersen S. Kringlen E. Cramer V. (2001). The prevalence of personality disorders in a community sample. Archives of General Psychiatry, 58(6), 590–596. 11386989 10.1001/archpsyc.58.6.590

[bibr96-00332941211027322] Twenge J. M. Konrath S. Bushman B. J. Foster J. D. Campbell W. K. (2008). Egos inflating over time: A cross-temporal Meta-analysis of the narcissistic personality inventory. Journal of Personality, 76(4), 875–902. 18507710 10.1111/j.1467-6494.2008.00507.x

[bibr321-00332941211027322] Velotti P. Elison J. Garofalo C. (2014). Shame and aggression: Different trajectories and implications. Aggression and Violent Behavior, 19(4), 454–461.

[bibr101-00332941211027322] Vernon P. A. Villani V. C. Vickers L. C. Harris J. A. (2008). A behavioral genetic investigation of the dark triad and the big 5. Personality and Individual Differences, 44(2), 445–452.

[bibr322-00332941211027322] Wallace M. T. Barry C. T. Zeigler-Hill V. Green B. A. (2012). Locus of control as a contributing factor in the relation between self-perception and adolescent aggression. Aggressive Behavior, 38, 3, 213–221. 10.1002/ab.21419 22531997

[bibr102-00332941211027322] Warren J. I. Burnette M. South S. C. Chauhan P. Bale R. Friend R. (2002). Personality disorders and violence among female prison inmates. The Journal of the American Academy of Psychiatry and the Law, 30(4), 502–509. 12539904

[bibr103-00332941211027322] Watson P. J. Biderman M. D. Boyd C. (1989). Androgyny as synthetic narcissism: Sex role measures and kohut's psychology of the self. Sex Roles, 21(3–4), 175–207.

[bibr104-00332941211027322] Watson P. J. Taylor D. Morris R. J. (1987). Narcissism, sex roles, and self-functioning. Sex Roles, 16(7–8), 335–350.

[bibr105-00332941211027322] Webster G. D. (2005). Low Self-Esteem is related to aggression, but especially when controlling for gender: A replication and extension of Donnellan et al. Representative Research in Social Psychology, 29, 12–18. 2006).

[bibr106-00332941211027322] Webster G. D. Kirkpatrick L. A. Nezlek J. B. Smith C. V. Paddock E. L. (2007). Different slopes for different folks: Self-esteem instability and gender as moderators of the relationship between self-esteem and attitudinal aggression. Self and Identity, 6(1), 74–94.

[bibr107-00332941211027322] Widiger T. A. Costa P. T. (2013). Personality disorders and the five-factor model of personality. American Psychological Association.

[bibr217-00332941211027322] Wink P. Gough H. G. (1990). New Narcissism Scales for the California Psychological Inventory and MMPI. Journal of Personality Assessment. 54, 446–462. 2348334 10.1080/00223891.1990.9674010

[bibr108-00332941211027322] Wolven K. E. (2015). *Grandiose and vulnerable narcissism: Where do the emotional differences lie?* (Psychology Theses Paper 18). USC Aiken.

[bibr434-00332941211027322] Wright A. G. C. (2014). Integrating trait and process based conceptualizations of pathological narcissism in the DSM-5 era. In Besser A. (Ed.), Handbook of psychology of narcissism: Diverse perspectives (pp. 153–174). Hauppauge, NY: Nova Science.

[bibr109-00332941211027322] Wright A. G. C. Lukowitsky M. Pincus A. Conroy D. (2010). The higher order factor structure and gender invariance of the pathological narcissism inventory. Assessment, 17(4), 467–483. 20634422 10.1177/1073191110373227

[bibr110-00332941211027322] Wright A. G. C. Pincus A. L. Thomas K. M. Hopwood C. J. Markon K. E. Krueger R. F. (2013). Conceptions of narcissism and the DSM-5 pathological personality traits. Assessment, 20(3), 339–352. 23610234 10.1177/1073191113486692

[bibr111-00332941211027322] Zeigler-Hill V. Clark C. B. Pickard J. D. (2008). Narcissistic subtypes and contingent self-esteem: Do all narcissists base their self-esteem on the same domains? Journal of Personality, 76(4), 753–774. 18482357 10.1111/j.1467-6494.2008.00503.x

[bibr112-00332941211027322] Zerach G. (2016). Pathological narcissism, cyberbullying victimization and offending among homosexual and heterosexual participants in online dating websites. Computers in Human Behavior, 57, 292–299.

